# A Pilot Test of the Measures of the Greek Version of Upper Extremity Functional Index in Patients with Lateral Elbow Tendinopathy

**DOI:** 10.3390/medsci11030045

**Published:** 2023-06-28

**Authors:** Eleftherios Paraskevopoulos, George Plakoutsis, Maria Papandreou

**Affiliations:** 1 Department of Physiotherapy, University of West Attica, 12243 Athens, Greece; 2 Department of Physiotherapy, University of Peloponnese, 23100 Sparta, Greece

**Keywords:** function, lateral elbow tendinopathy, upper extremity functional index, measurement property, cross-cultural adaptation

## Abstract

Lateral elbow tendinopathy (LET) is a common upper limb pathology in people involved in manual occupations. The upper extremity functional index (UEFI) was specifically designed to evaluate functional limitations in patients with upper limb pathology. The UEFI was developed in English and has been translated into several languages, including Greek. However, it has been assessed only in patients with shoulder pathology. Thus, the aim of this study was to pilot-test the Greek version of the UEFI (GV-UEFI) questionnaire and assess its measurement properties in patients with LET. Thirty patients with LET were recruited and asked to fill in the GV-UEFI twice and the disabilities of arm, shoulder, and hand questionnaire (DASH) once. The internal consistency and test–retest reliability were examined using Cronbach’s alpha and the intraclass correlation coefficient (ICC). The standard error of measurement (SEM) and the minimum detectable change (MDC) were calculated and possible ground or ceiling effects were also examined. Convergent validity was evaluated with the Greek DASH using Pearson’s correlation. Lastly, the unidimensionality of the scale was examined through principal component analysis to verify construct validity. Internal consistency was high for the GV-UEFI (Cronbach’s a = 0.98) and test–retest reliability was excellent (ICC = 0.98). The SEM was 2.95 and the MDC was 6.85. Test–retest reliability of each item was good (ICC > 0.87). The correlation analysis demonstrated a strong correlation between the GV-UEFI and the DASH. No floor or ceiling effects were found. Principal component analysis verified the construct validity and the unidimensionality of the scale. The GV-UEFI was successfully tested in patients with LET. It seems that the GV-UEFI can be used reliably in Greek-speaking patients with LET. However, the measurement properties of this scale should be examined in a larger sample of LET patients.

## 1. Introduction

Lateral elbow tendinopathy (LET) is one of the most common pathologies of the upper limb in people involved in manual occupations characterised by repetitive movements of the arm (i.e., those in the construction industry and in manufacturing) and certain athletes, including tennis players [1]. LET occurs in up to 3% of the general population between the age of 30 and 60 years old [2]. LET has been described as a non-inflammatory condition that causes pain at the tendon insertion or the junction of the myotendon of the wrist extensors muscles. Patients with LET frequently report pain at the lateral epicondyle that negatively affects their function when using their affected limb [3].

Since LET can restrict patients and especially athletes in their activities of daily living and sport participation, an important aspect that should be assessed by clinicians is function. The function is usually assessed as part of the physiotherapy assessment as a prognostic criterion, but it can also help to understand whether any treatment used is beneficial for the patient [1]. The use of questionnaires in the management of patients suffering from upper limb pathologies is essential for therapeutic monitoring [1]. Moreover, functional status has been correlated with progression of the tendinopathy which further highlights the need for a high-quality scale that evaluates function in this population [1]. Stratford [4] developed a questionnaire that was designed to examine patients’ upper limb functional status. The upper extremity functional index (UEFI) lists 20 functional activities and a possible score from 0 (extreme difficulty) to 4 (no difficulty) for each activity. Scores are given based on the level of difficulty when performing each one of these activities. The sum of the scores from each question is calculated, with a possible range of scores from 0 to 80. Clearly, greater scores indicate a better function for the patient.

The UEFI has shown excellent measurement properties in people with upper limb musculoskeletal pathologies [5,6,7,8]. These findings suggest that the UEFI should be used to effectively measure activity limitations in patients with upper limb pathology. The main advantage of the UEFI is that it is a region-specific questionnaire and it examines only activity limitation. Thus, a change in the UEFI final score can be interpreted as an improvement or a reduction in functional ability. The disabilities of the arm, shoulder and hand (DASH) or the patient-rated tennis elbow evaluation questionnaire (PRTEE) have shown acceptable responsiveness in patients with LET [1] and in patients surgically treated from LET [9]. The UEFI has similar responsiveness to the DASH, however, a change in either the DASH or the PRTEE score can be interpreted also as a change in symptom severity [10].

The UEFI has been translated and cross-culturally adapted into Greek, Chinese, Arabic and Turkish languages for patients with shoulder pathology [7,8,10,11]. However, this questionnaire has not been validated in patients with LET. Validating the UEFI in patients with LET will allow clinicians to use it in clinical practice, since LET patients have demonstrated significant functional restrictions [3]. We understand that the use of the UEFI is not restricted only in patients with LET or shoulder pathology since it is a scale that assesses upper limb functional status, however, research suggests that the properties of patient-reported outcome measures (PROM) should be examined across different patient populations, treatments, and contexts to fully understand the performance of PROM [12]. Thus, the aim of our study was to pilot-test the UEFI in Greek language and examine the internal consistency, test–retest reliability, convergent validity, construct validity and floor–ceiling effects among patients with LET.

## 2. Materials and Methods

Convenience sampling was used to recruit participants with LET. To be eligible to participate in the study, patients had to meet the following criteria: (1) be adults (>18 years), (2) have a diagnosis of unilateral LET (lateral epicondylalgia or tennis elbow diagnosis were also included under the umbrella term LET) and (3) be able to natively communicate, read and write in Greek. The diagnosis was done based on a physical examination. Patients satisfied the inclusion criteria if they complained for pain over the lateral epicondyle (and/or 1–2 cm distal to epicondyle) and had pain and weakness on resisted wrist extension, testing positive on the Cozen test and also if they complained for pain when they passively stretched their wrist in flexion, testing positive in the Mill’s test [1]. Patients were excluded if they had cognitive, communication, or psychological issues or if they had been surgically treated for musculoskeletal problems, or if they were suffering from neurological dysfunction or cardiovascular or pulmonary dysfunctions that were rated by the patient as functionally limiting. All participants signed an informed consent form prior to participation. The study protocol was approved by the Ethics Committee of the University of the Peloponnese (Registration number: 2289).

### 2.1. Sample Size

A minimum sample of 30 participants was considered fair for the assessment of the internal consistency, floor and ceiling effects, convergent validity, test–retest reliability, measurement error, and construct validity based on the recommendation of the consensus-based standards for the selection of health measurement instruments (COSMIN) [13].

### 2.2. Greek Version of the UEFI (GV-UEFI)

The translated UEFI was used based on a previous study that completed the procedures of translation and cross-cultural adaptation in patients with rotator cuff-related shoulder pain [8] using established international guidelines [14]. The GV-UEFI was initially tested in 2 Greek patients with upper extremity musculoskeletal pathologies. After the completion of the GV-UEFI by these 2 patients, an interview followed. Two physiotherapists with more than 10 years of clinical experience conducted the interviews. In each individual interview, the participants were asked to explain if the content of the GV-UEFI was clear and related to elbow pathology after reading the instructions, the items and the responses. Furthermore, they were asked to clarify whether parts of the scale were not clear and to provide suggestions for any possible modifications that could have improved clarity. The entire scale was found to be well conceivable by all patients and appropriate for patients with elbow pathology.

### 2.3. Reliability

Test–retest reliability was examined in the final Greek version of the UEFI (GV-UEFI). All participants (*n* = 30) were asked to complete the GV-UEFI twice. The first time that they completed the GV-UEFI was during their first contact with the authors and the second was 3 to 5 days later. The interval of test–retest sessions in this study was specified in order to minimise recall and to be suitably short to guarantee clinical stability between testing sessions [10]. Clinical stability of the participants was examined by asking each participant whether they believed that their symptoms were the same in the retest session. Only patients that answered that their symptoms were the same in the retest session completed the GV-UEFI for a second time. Completion of the GV-UEFI twice allowed the investigators to examine test–retest reliability by comparing the results of the test and retest sessions. Internal consistency was also assessed based on the degree to which separate items of the GV-UEFI related to each other [15].

### 2.4. Validity

The convergent validity for patients with LET was examined after correlating the results of the GV-UEFI with the Greek version of the disabilities of the arm, shoulder, and hand (DASH) [10]. The Greek DASH has shown to be a reliable and valid instrument that can provide a standardised measure of patient-centred outcomes in Greek-speaking patients with upper limb disorders [16]. The DASH contains 30 questions; 21 related to function, 6 related to symptom severity and 3 to social function. Each question is rated on a 5-point scale (1, no difficulty; 2, mild difficulty; 3, moderate difficulty; 4, severe difficulty; and 5, unable). The questionnaire score is calculated by applying established formulas for the first 30 questions and the scores range from 0 (the best) to 100 (the worst) [16]. The authors of this study were aware that the DASH examines symptom severity, which is not included in the GV-UEFI. However, it was decided to correlate the results of the GV-UEFI with the DASH since an upper extremity region-specific outcome measure, quantifying only activity limitation, was not available in the Greek language, as previously suggested [10].

The DASH was chosen to allow the authors to generate a priori hypotheses related to expected correlations of the GV-UEFI with the DASH. A strong correlation (>0.60) was expected between the GV-UEFI and the DASH. The correlation of the GV-UEFI with the sub-scales of the DASH (function, severity of symptoms and social function) was also examined. The sub-scales of the DASH were established based on previous research [16]. Questions 1–21 were related to physical function, 22–27 to symptom severity and 28–30 to social function. As stated above, the UEFI examines upper limb function and thus, a unidimensional structure is expected that allows a single valid summated score to be achieved. To confirm this, principal component analysis was conducted to verify unidimensionality and construct validity.

### 2.5. Statistical Analysis

All data were analysed using IBM SPSS statistics 25.0. The statistical level of significance was set at *p* < 0.05. The normal distribution of the data was examined through visual inspection of the Q–Q plots.

#### 2.5.1. Internal Consistency and Test–Retest Reliability (Measurement Errors)

The internal consistency of the GV-UEFI was examined using Cronbach’s alpha and it was calculated from the pairwise correlations between items of the scale. This analysis was also conducted for the retest session. Internal consistency was considered acceptable for the GV-UEFI if Cronbach’s alpha value was within the recommended range of 0.70–0.95 [17]. The intraclass correlation coefficient (ICC) for absolute agreement was used to examine test–retest reliability of each item and the total score of the GV-UEFI. Measurement errors from the use of GV-UEFI were estimated from the standard error of measurement (SEM) and the minimum detectable change (MDC90). For the SEM, the following equation was used: SEM = SD√(1-ICC). SD was the pooled SD calculated using the following equation: SDpooled = √(SD1^2^ + SD2^2^)/2. Then, the MDC90 was calculated using the following equation: MDC90 = SEM × 1.64 × √ 2 [18,19].

#### 2.5.2. Convergent Validity

Convergent validity of the GV-UEFI was examined by correlating the total scores of the GV-UEFI with the total scores of the Greek DASH. Pearson’s correlation was used to examine convergent validity based on our predefined hypothesis suggesting that at least a correlation of >0.60 will be evident between the GV-UEFI and the DASH [17]. Since the scoring scale of the DASH is in the opposite direction (lower scores indicate that the person is reporting reduced symptom severity) compared to the UEFI, we were expecting a negative correlation. Using the recommendations of a previous study that used the same instruments, we hypothesised that a correlation value of ≥ 0.60 indicates a strong correlation, a value between 0.40 and 0.59 a moderate correlation, and a value ≤ 0.39 indicates a weak correlation between the questionnaires [7].

#### 2.5.3. Floor and Ceiling Effects

Verification of floor and ceiling effect was performed based on the percentage (>15%) of the participants who have obtained the minimum and maximum scores in the GV-UEFI [20].

#### 2.5.4. Construct Validity

A principal component analysis was performed to verify the factor structure of the GV-UEFI in patients with LET. Extraction and retention of factor structure was performed after inspection of the scree plot. Eigenvalues of > 1.0 were retained.

## 3. Results

The GV-UEFI was completed twice by 17 men and 13 women with LET with a mean age of 47.4 (± 9.4, range 20–58) and a duration of symptoms of 4.1 (± 1.2, range 1–6) months. From this sample of 30 patients, 24 had LET on the right side and the rest on the left. All patients were taking over-the-counter oral analgesics and/or anti-inflammatories for symptom reduction. The mean score of the GV-UEFI in the first assessment was 38.8 (±12, range 20–60) and at retest was 38.3 (±11.8, range 20–60). The mean score of the DASH was 51.6 (±15.5, range 20–74) (Table 1). Data seemed to be normally distributed based on the visual inspection of the Q–Q plots.

### 3.1. Internal Consistency and Test–Retest Reliability

Internal consistency for patients with LET was high for the GV-UEFI in both the test (Cronbach’s a = 0.978) and retest sessions (Cronbach’s a = 0.974). Pearson’s correlation between each item and the sum of all the other items was evaluated from the “Corrected Item-Total Correlation” of the first test session to assess whether all items are measuring the same underlying construct [21]. Indeed, the analysis did not reveal any correlation coefficients lower than 0.3, indicating that all items were measuring the same underlying construct. ICC was 0.98 (95% confidence interval (CI): 0.98, 0.99) for the GV-UEFI indicating an excellent test–retest reliability. The SEM was 2.95 and the MDC was 6.85. Test–retest reliability of each item was good (ICC > 0.87), as shown in Table 2.

### 3.2. Validity

Correlating the GV-UEFI completed in the first assessment with the DASH allowed us to examine the convergent validity of the GV-UEFI for patients with LET. Indeed, after analysing the data from the DASH we found a significant strong correlation (r = −0.744, and *p* < 0.001). Moreover, correlating the GV-UEFI with the subscales of the DASH confirmed our initial hypothesis of a strong correlation, as shown in Table 3.

### 3.3. Floor or Ceiling Effects

Considering that the highest possible score of the UEFI is 80 and the lowest possible score is 0, we examined the total scale as a single dimension. The examination of the total scale revealed no floor or ceiling effects in our sample.

### 3.4. Construct Validity

**Figure 1:**
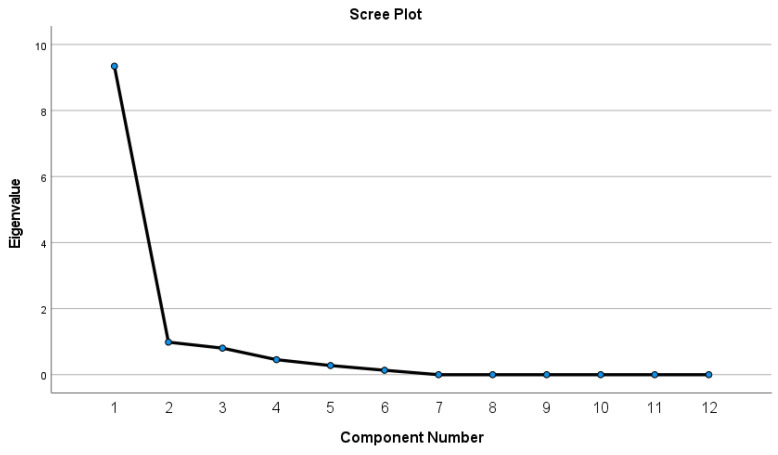
Scree plot of the principal component analysis.

From the principal component analysis, the unidimensionality of the GV-UEFI was verified, since only one component was revealed with an eigenvalue greater than 1 (component 1 = 9.34) that explained 78% of the total variance. The scree plot further verified the unidimensionality of the GV-UEFI and clearly indicated that one component should be retained (Figure 1). However, it should be mentioned that due to low sample size, the assumptions of the principal component analysis (Kaiser–Meyer–Olkin and Bartlett’s test of sphericity) were not met, indicating that the results of this study should be interpreted with caution and that analysis of a larger sample size is needed to draw strong conclusions.

## 4. Discussion

The findings of this study supported our initial hypothesis that the GV-UEFI will have excellent internal consistency, test–retest reliability and convergent validity when correlated with the DASH questionnaire in patients with LET. Furthermore, the GV-UEFI had an acceptable measurement error without any floor or ceiling effects, indicating that it is a valid questionnaire for assessing upper limb function in patients with LET. Moreover, unidimensionality was verified in this small sample of patients through principal component analysis; however, a larger sample is needed for strong conclusions to be drawn.

From the assessment of the internal consistency, Cronbach’s alpha was found to be higher than the recommended range, indicating that some of the items in the GV-UEFI may be redundant [10]. However, with the removal of one question at a time using the Cronbach’s alpha if item deleted option from SPSS, the Cronbach’s alpha remained high (0.98), indicating that none of the items were redundant. Furthermore, Pearson’s correlation between each question of the GV-UEFI and the sum of all the other items ranged from 0.33 to 0.97, indicating that the questionnaire could differentiate between participants in the construct measured when evaluating upper limb function in patients with LET [22] (Table 2). Lastly, the reported values of internal consistency so far in the literature range from 0.89 to 0.96 [4,7,8,10,11,23] which are fairly close to the internal consistency value found in our study.

The test–retest reliability in patients with LET was examined using the ICC. The GV-UEFI showed excellent test–retest reliability with an ICC value of 0.98 (95% confidence interval (CI): 0.98, 0.99). This finding suggests that the GV-UEFI is stable over time when used for patients with LET [10]. Moreover, the 95% CI was in the recommended threshold valued of >0.70, indicating that the GV-UEFI is also reliable [24]. In fact, this study showed that the GV-UEFI has the highest 95% CI when compared with other studies using the UEFI in patients with shoulder pathology [7,10,11].

Moreover, the SEM and the MDC were 2.95 and 6.85, respectively. These findings suggest that when using the GV-UEFI to detect a true change in upper limb function in patients with LET, a difference of ≥6.85 should be found between sessions. These findings are in line with almost all studies reporting the SEM (2.2–4.9) and the MDC (8.1–17.6) values when evaluating the UEFI in other languages [7,8,10,11]. Although the MDC is slightly lower than the range of values from previous studies, we believe that, SEM and MDC demonstrated acceptable measurement errors. Of course, it should be noted that a range of factors may affect the measurement error, including the duration between test–retest assessment, the age of the participants and the severity of symptoms in LET as compared to other clinical populations [1,2,25]. Thus, direct comparison between studies is sometimes impractical.

Our initial hypothesis was that the GV-UEFI will have a strong correlation with the DASH in patients with LET, although the UEFI is solely a scale that evaluates activity limitation in patients with musculoskeletal pathologies. The findings from the statistical analysis confirmed our hypothesis. This finding is in agreement with the findings of other studies evaluating the validity of the UEFI with the patient specific functional scale (PSFS), the upper extremity functional scale (UEFS), the shoulder pain and disability index (SPADI) and the Constant–Murley Score (CMS) [10,26,27,28,29], which further support the results of our study. Furthermore, similar studies performed using the cultural adaptation of the UEFI in other languages (Arabic, Turkish, and Chinese) and in Greek in patients with shoulder pathology have reported correlation values between the UEFI and the DASH that ranged from 0.62 to 0.95 [7,8,10,11]. The results of these studies are in line with our findings, since the correlation value that we found in this study (r = 0.744) is within the reported range found in the aforementioned studies.

Verification of the ground and ceiling effect was performed based on the percentage (>15%) of the participants who obtained the minimum and maximum scores in the GV-UEFI. Interestingly, no ground or ceiling effects were evident in our sample. This finding suggests that the GV-UEFI can appropriately examine functional limitations in patients with LET, with symptom duration up to six months [30]. Also, the lack of ground or ceiling effects demonstrates that the GV-UEFI has good content validity [30].

Research suggests that the UEFI has a single dimension structure that allows clinicians to reliably examine only upper limb function. This was verified in this study from the results of the principal component analysis [26]. However, it should be mentioned that since the assumptions of this test were not met due to a small sample size, this finding should be treated with caution and this study should be considered as a pilot study.

While this pilot study successfully examined the GV-UEFI in patients with LET, there were some limitations that should be considered. Firstly, the responsiveness of the GV-UEFI was not measured. Also, subgroup comparisons were not performed to evaluate whether differences between different age groups, or genders, or pain severity levels may exist. Of course, the number of our sample (*n* = 30) did not allow the authors to perform such comparisons, highlighting the need for a large-scale study. Moreover, a similar study with a larger sample size will allow the investigators to accurately examine the unidimensionality of the GV-UEFI and verify its construct validity. Based on the recommendation of the consensus-based standards for the selection of health measurement instruments (COSMIN), a minimum sample of 100 patients is considered as an excellent sample size in order to make strong and valid conclusions [13]. Thus, a large-scale study will allow generalisability of the study results in patients with LET. Another aspect that may be seen as a limitation is the fact that we only asked participants to indicate whether their severity of symptoms remained constant, without actually grading pain levels with a scale such as the visual analogue scale. Grading pain levels with a scale would have reassured all authors about the clinical stability of the participants between test–retest sessions. However, the small timeframe between test–retest assessment reduced the chance for substantial changes to occur.

This study recruited patients with non-chronic LET who may experience different signs and symptoms when compared with patients with chronic LET. Moreover, research has shown that the tissue of patients with chronic LET has no inflammatory cells (macrophages, lymphocytes, and neutrophils) and is often characterised by degeneration and angiofibroblastic hyperplasia [31]. Furthermore, patients with chronic LET are more likely to suffer from central sensitisation with widespread mechanical pain hypersensitivity which could influence the performance of PROM when compared with patients with non-chronic LET [32]. Moreover, this study recruited patients with a mean age of 47.4 since studies have shown that patients between 30 and 50 years are more likely to suffer from LET [33,34]. However, since the UEFI is a PROM that examines functional status and it is not a disease-specific questionnaire, the findings of this study fully support the use of the GV-UEFI in all patients with LET. In future studies, patients with chronic LET may be also recruited since PROMs need to be examined across different patient populations, treatments, and contexts to fully understand their performance [12]. Our suggestions of the examination of the GV-UEFI in other clinical populations is based on previous reports suggesting that in order to identify issues that are specific to a patient and to assess change over time, consideration of the outcome of interest and the conditions specific to a patient group should be conducted [26].

## 5. Conclusions

In this study, the GV-UEFI was pilot-tested in patients with LET. The GV-UEFI showed high internal consistency, excellent test–retest reliability, acceptable measurement error and convergent validity and no ground or ceiling effects in this population. Moreover, the GV-UEFI demonstrated similar measurement properties with other translations and with the original version. Thus, the GV-UEFI can be used by Greek-speaking patients with LET either for clinical or research purposes, however, a larger-scale study should be performed to verify the unidimensionality of the GV-UEFI and ensure generalizability in this clinical population.

## Figures and Tables

**Table 1:** Participants’ characteristics (*n* = 30).

Characteristics	Mean	Standard Deviation ±
Age (years)	47.4	9.4
Symptom duration (months)	4.1	1.2
GV-UEFI score (1st assessment)	38.8	12
GV-UEFI score (2nd assessment)	38.3	11.8
DASH score	51.6	15.5

Abbreviations: GV-UEFI, Greek version of the upper extremity functional index; DASH, disabilities of arm, shoulder and hand questionnaire.

**Table 2:** Test–retest reliability of each item of the GV-UEFI.

Questions	ICC	Cronbach’s Alpha If Item Deleted	Corrected Item-Total Correlation
Any of your usual work, housework, or school activities	0.93	0.988	0.978
Your usual hobbies, recreational or sporting activities	0.97	0.988	0.978
Lifting a bag of groceries to waist level	0.97	0.988	0.755
Lifting a bag of groceries above your head	1	0.988	0.888
Grooming your hair	0.96	0.988	0.887
Pushing up on your hands (e.g., from bathtub or chair)	0.93	0.988	0.964
Preparing food (e.g., peeling, and cutting)	0.96	0.988	0.422
Driving	1	0.988	0.978
Vacuuming, sweeping or raking	1	0.988	0.964
Dressing	0.97	0.988	0.612
Doing up buttons	0.97	0.988	0.978
Using tools or appliances	0.97	0.988	0.888
Opening doors	0.96	0.988	0.978
Cleaning	0.93	0.988	0.773
Tying or lacing shoes	0.93	0.988	0.892
Sleeping	0.96	0.988	0.964
Laundering clothes (e.g., washing, ironing, and folding)	1	0.988	0.731
Opening a jar	0.92	0.988	0.336
Throwing a ball	0.96	0.988	0.706
Carrying a small suitcase with your affected limb	0.87	0.988	0.949

Abbreviations: ICC: Intraclass correlation coefficient between test–retest for each question, Cronbach’s alpha if item deleted: how the calculated Cronbach’s alpha value would change when each specific item is removed from the scale, Corrected item-total correlation: Pearson’s correlation between the specific item and the sum of all the other items.

**Table 3:** Correlation between GV-UEFI and DASH.

Scales	Correlation (r)	Significance
GV-UEFI—DASH	0.744	*p* < 0.001
GV-UEFI—DASH-Function	0.690	*p* < 0.001
GV-UEFI—DASH-Symptom severity	0.753	*p* < 0.001
GV-UEFI—DASH-Social function	0.663	*p* < 0.001

Abbreviations: GV-UEFI, Greek version of the upper extremity functional index; DASH, disabilities of arm, shoulder and hand questionnaire.

## Data Availability

The data presented in this study are available on request from the corresponding author. The data are not publicly available due to ethical issues.
